# Social Networks and Knowledge Transmission Strategies among Baka Children, Southeastern Cameroon

**DOI:** 10.1007/s12110-018-9328-0

**Published:** 2018-10-24

**Authors:** Sandrine Gallois, Miranda J. Lubbers, Barry Hewlett, Victoria Reyes-García

**Affiliations:** 1 Faculty of Archaeology, Leiden University, Einsteinweg 2, 2333 Leiden, CC Netherlands; 2 Departament d’Antropologia Social i Cultural, Universitat Autònoma de Barcelona, 08193 Bellaterra, Spain; 3 Department of Anthropology, Washington State University, Vancouver, WA USA; 4 Institut de Ciència i Tecnologia Ambientals, Universitat Autònoma de Barcelona, 08193 Bellaterra, Spain; 5 Institució Catalana de Recerca i Estudis Avançats, Pg. Lluís Companys 23, 08010 Barcelona, Spain

**Keywords:** Cultural transmission, Ethnoecology, Hunter-gatherers, Subsistence activities, Social learning

## Abstract

The dynamics of knowledge transmission and acquisition, or how different aspects of culture are passed from one individual to another and how they are acquired and embodied by individuals, are central to understanding cultural evolution. In small-scale societies, cultural knowledge is largely acquired early in life through observation, imitation, and other forms of social learning embedded in daily experiences. However, little is known about the pathways through which such knowledge is transmitted, especially during middle childhood and adolescence. This study presents new empirical data on cultural knowledge transmission during childhood. Data were collected among the Baka, a forager-farmer society in southeastern Cameroon. We conducted structured interviews with children between 5 and 16 years of age (*n* = 58 children; 177 interviews, with children being interviewed 1–6 times) about group composition during subsistence activities. Children’s groups were generally diverse, although children tended to perform subsistence activities primarily without adults and with same-sex companions. Group composition varied from one subsistence activity to another, which suggests that the flow of knowledge might also vary according to the activity performed. Analysis of the social composition of children’s subsistence groups shows that vertical and oblique transmission of subsistence-related knowledge might not be predominant during middle childhood and adolescence. Rather, horizontal transmission appears to be the most common knowledge transmission strategy used by Baka children during middle childhood and adolescence, highlighting the importance of other children in the transmission of knowledge.

**Electronic supplementary material:**

The online version of this article (10.1007/s12110-018-9328-0) contains supplementary material, which is available to authorized users.

The dynamics of knowledge transmission and acquisition, or how different aspects of culture are passed from one individual to another and how they are acquired and embodied by individuals, are central to understanding cultural evolution (Guglielmino et al. 1995; Henrich and Broesch 2011; Hewlett and Cavalli-Sforza 1986). Evolutionary theories and mathematical models have identified several pathways through which knowledge is transmitted: vertical (from parents and grandparents to children), oblique (from adults other than parents or grandparents to younger generations), and horizontal (between age peers) (Cavalli-Sforza et al. 1982; McElreath and Strimling 2008). Understanding which pathway predominates is important because different pathways have different impacts on the conservation of cultural traits and, therefore, cultural evolution. For example, vertical transmission enhances cultural continuity but limits innovations (Cavalli-Sforza et al. 1982; McElreath and Strimling 2008), whereas oblique and horizontal transmission can lead to rapid diffusion of new cultural traits, especially if contact with transmitters is frequent (Hewlett and Cavalli-Sforza 1986; Hewlett et al. 2011; Reyes-García et al. 2009).

The long juvenile period unique to humans is a key stage for the acquisition of knowledge that will be needed during adulthood (Kaplan et al. 2000). Indeed, as demonstrated by previous research in small-scale societies, the acquisition of cultural knowledge, including culturally shaped ecological knowledge and social norms, begins early in life and progresses quickly; by 12 years of age, most children have already accumulated large amounts of cultural knowledge (e.g., Demps et al. 2012; Gallois et al. 2017; Gurven et al. 2006; Hunn 2002; Zarger and Stepp 2004). However, the process of knowledge acquisition can vary according to the knowledge domain. For example, whereas young adolescents might hold knowledge about wild edibles similar to that of adults (Gallois et al. 2017), the same is not true for hunting or medicinal plants (Gurven et al. 2006; Hill and Hurtado 1996) since only adults can master such domains of knowledge.

The pathways of cultural transmission that occur during childhood are thought to have a considerable impact on the process of cultural evolution. In small-scale societies, children’s acquisition of cultural knowledge is said to mostly rely on observation, imitation, teaching, and participation in daily activities (Gaskins and Paradise 2010; Hewlett et al. 2011; Rogoff et al. 2007). Moreover, previous research in small-scale societies has also advanced our knowledge of the predominance of different pathways for cultural knowledge transmission during childhood, suggesting that the relative importance of each of the different pathways depends on the learner’s stage in the life cycle (Bird and Bliege Bird 2002; Demps et al. 2012; Gurven and Kaplan 2006; Haselmair et al. 2014; Ingold 2010; Kline et al. 2013; Rogoff et al. 2007). Thus, whereas vertical transmission seems to be important during infancy and early childhood, horizontal and oblique transmission gain importance during middle childhood (Boyette 2013; Garfield et al. 2016; Hewlett et al. 2011). Indeed, children might sequentially or simultaneously use different knowledge transmission pathways to update their cultural knowledge in what has been named a “multiple-stage learning process” (Reyes-García et al. 2016; Schniter et al. 2015).

Recent studies also point to important gaps in research on cultural knowledge acquisition. Thus, although we know that oblique and horizontal transmission become important once children enter middle childhood (Hewlett 2014a), we know less about how these pathways operate during middle childhood and adolescence (see Aunger 2000; Demps et al. 2012; Kline et al. 2013 for exceptions). Moreover, previous research has typically addressed only one domain of knowledge (e.g., medicinal plants or hunting), but recent work has highlighted that the transmission of cultural traits might vary according to the domain (Garfield et al. 2016; Salali et al. 2016; Zent 2013), so findings from one domain might not be applicable to others. Finally, although we know that observation and imitation are important for knowledge acquisition, few researchers have focused on understanding who children observe and imitate (Gurven and Kaplan 2006; Haselmair et al. 2014; Ingold 2010). Given that cultural transmission is affected by context and content biases (Broesch et al. 2014; Salali et al. 2016), we propose that documenting group composition during the performance of subsistence activities is a promising venue for understanding the process of cultural transmission, specifically contributing to identification of children’s role models (Henrich and Broesch 2011). For example, previous scholars have shown that knowledge transmission might be shaped by homophily—the social preference to form same-sex or same-age groups (Acerbi and Alexander 2014; Diaz-Reviriego et al. 2017; Wood et al. 2013), or by the tendency to imitate high-prestige individuals (Henrich and Gil-White 2001; Henrich and McElreath 2003; Richerson and Boyd 2005). Thus, determining whom children learn from or imitate might contribute to our knowledge of cultural transmission.

In this article, we explore children’s group composition during the performance of subsistence activities using primary data collected among the Baka of southeastern Cameroon. We measured group composition using social network analysis because, in other settings and contexts, this research tool has proven to be useful to assess the influence of group structure on knowledge transmission (Henrich and Broesch 2011; Salali et al. 2016; Subramani and Rajagopalan 2003), with some recent research analyzing how social networks shape the flow of unwritten knowledge (Calvet-Mir et al. 2012; Hamilton et al. 2007; Hopkins 2011; Migliano et al. 2017; Salpeteur et al. 2015). Specifically, we focus on children’s group composition during the performance of four subsistence activities (hunting, gathering, fishing, and agriculture) and consider the implications that group composition may have on children’s knowledge transmission. Our work assumes that people who perform activities together share information and knowledge related to the activity (Migliano et al. 2017; Morelli et al. 2003). We are aware that performing an activity in a group does not imply that all the people involved in it interact or share knowledge in the same way; nevertheless we consider that the analysis of group composition might provide clues about children’s potential learning models (Henrich and Broesch 2011).

Our work has three goals. First, we aim to evaluate whether the way in which knowledge might flow varies depending on the type of activity. To do so, we analyze group size and composition while children perform subsistence activities. Baka adults perform some subsistence activities in groups (e.g., fishing, gathering) and others alone (e.g., hunting). Observation and imitation are important learning strategies used by children to reproduce adults’ daily activities, and previous work suggests that Baka children imitate different aspects of adult behavior, from tools to sexual division of labor (Gallois et al. 2015). Therefore, we expect children’s group composition to resemble that of adults.

Our second goal is to evaluate whether the patterns of possible knowledge transmission vary with children’s age. To do so, we first evaluate the likelihood of vertical, oblique, and horizontal transmission pathways during children’s performance of subsistence activities in different stages of childhood. Drawing on previous literature (Garfield et al. 2016; Hewlett et al. 2011), we expect to observe a decreasing predominance of vertical transmission and an increasing predominance of oblique and horizontal transmission pathways with age.

Our third goal is to evaluate whether children’s performance of subsistence activities shows gender- or kinship-related biases. Based on previous research showing gender differentiation in children’s activities (Gallois et al. 2015), we expect to find gender homophily in children’s groups. Moreover, because kinship relations impact social daily life among hunter-gatherer children (Hewlett 2014b), we expect to find relatively high numbers of kin in children’s groups.

## The Baka

The Baka are a society of about 30,000 individuals living in the tropical forest of the Congo Basin. Their territory spreads across the Republic of Congo, Gabon, Central African Republic, and Cameroon (Joiris 1998; Leclerc 2012). Traditionally, the Baka were a nomadic society, mostly subsisting on the products of foraging and on barter with their neighbors, sedentary Bantu-speaking farmers. In southeastern Cameroon, where this study took place, the Baka have experienced several changes since the 1950s, including a generalized shift to a more forager-farmer livelihood. Beginning in the 1950s, the increasing arrival of outsiders and the reduction of forest resources (especially game), together with missionaries and government programs, started to push the Baka to settle in permanent villages, mostly along logging roads (Bailey et al. 1992; Leclerc 2012). Today, the Baka primarily live in permanent settlements, moving to their forest camps during the season when they gather non-timber forest products for sale. While in the forest camps, the Baka generally live in small bands (around 30 people), a situation that contrasts with their lives in the village, where the population can reach into the hundreds.

Despite these changes, the Baka continue to be a highly egalitarian society characterized by respect for the autonomy of every individual (including children) and the extensive sharing of goods and services (Joiris 1998). Although the Baka continue to be highly dependent on forest resources, most of them now also engage in agricultural work. Involvement in agriculture varies between households, but for some households, farming, either in their own plots or providing casual labor to neighboring Bantu villages, has become a major economic activity (Gallois et al. 2016; Leclerc 2012).

As in other hunter-gatherer societies (Hewlett 2014a), Baka infants and small children benefit from allomaternal care provided by siblings or other family members (Hirasawa 2005). From middle childhood onward, children’s daily life revolves around the performance of household maintenance and subsistence activities and play (Gallois et al. 2015). As in other societies (Boyette 2016), subsistence activities are generally performed with enjoyment, and the border between what could be considered work and play is somewhat blurred. Adults’ and children’s activities are differentiated by gender: boys are more involved in hunting whereas girls are more involved in fishing and gathering (Gallois et al. 2015). Related to these subsistence activities, Baka children hold several types of ecological knowledge. We previously showed that while children hold similar knowledge as adults regarding wild edibles, their hunting-related knowledge was different from that of adults (Gallois et al. 2015). In addition to showing the progressive nature of learning such complex knowledge, we also highlighted that children hold their own specific knowledge that is not shared with adults.

## Methods

Our study took place in a Baka community in the Haut-Nyong Department, in the East Province of Cameroon, where the first author performed ethnographic fieldwork for 18 months (February 2012 to May 2014). The village had 264 individuals and is located 45 km from the main administrative town and 2 km from the nearest Bantu-speaking Nzime farmers’ village. The village was composed of four main hamlets, the farthest one about 200 m from the center of the village. More than half of the population (*n* = 146) was under 16 years of age and thus considered children for the purpose of this study. Baka in the village were involved in forest-related activities (hunting, gathering, fishing) on a daily basis and in agricultural activities less frequently (see Gallois et al. 2015 for details).

### Data Collection

Before the onset of the study, the first author obtained Free Prior and Informed Consent (FPIC) from every adult participant. For children, we asked for both parental and child’s consent. All data were collected in the context of immersion into Baka daily life: joining Baka in gathering and fishing trips and helping them with domestic work such as cooking or washing clothes. The first author learned the Baka language and adhered to social and cultural norms on sharing, cooking, and childcare as much as possible. Extensive fieldwork allowed her to conduct numerous informal conversations with both adults and children, male and female, and to gather ethnographic data about cultural transmission and acquisition as well as about the composition of children’s groups during daily activities.

To explore children’s group composition while performing subsistence activities, we conducted a census and structured interviews on children’s daily activities. The census collected information on the name, age, clan, and kinship relations of all people living in the village. Since most Baka cannot recall their date of birth and do not have birth records, we used kinship information to estimate children’s age. Specifically, we asked mothers to report children’s birth order and fixed at 2 years the birth interval among siblings (including miscarriage and deceased children) (Rozzi et al. 2015). We defined children as individuals between 5 and 16 years of age. Although cultural knowledge acquisition starts earlier in life, we set our lower limit at five years because younger children were too shy to answer interview questions. The upper limit was fixed at 16 years because at about this age young Baka typically form new households and are thus considered adults (Gallois et al. 2015).

To assess children’s group composition during daily activities, we conducted structured interviews with 58 children (26 girls and 32 boys), representing 61% of the children aged 5–16 in the village (*n* = 95). Fifteen informants were between 5 and 8 years old (middle childhood), 22 were between 9 and 12 (preadolescence), and 21 were between 13 and 16 (adolescents). Children in middle childhood were less well represented in the sample (38% of all children between 5 and 8 participated) than preadolescents (76% of the children in that age group) and adolescents (81% of them). Each interview lasted between 10 and 15 min. We first asked children whether they had engaged in hunting, gathering, fishing, or agriculture during the previous 24 h. Multiple reports of the same activity on a single day by the same child (i.e., hunting twice) were recorded as different observations. For each observation, we asked children to report the type of activity and the names of all the individuals who were with them while performing the activity. To avoid memory biases, we conducted most of the interviews at the end of the day. Most children were interviewed multiple times, so the total number of interviews is of 177 (101 among girls and 76 among boys). Two children were interviewed six times; 9, five times; 10, four times; 15, three times; 13, twice; and 9 were interviewed only once.

### Data Analysis

**Fig. 1:**
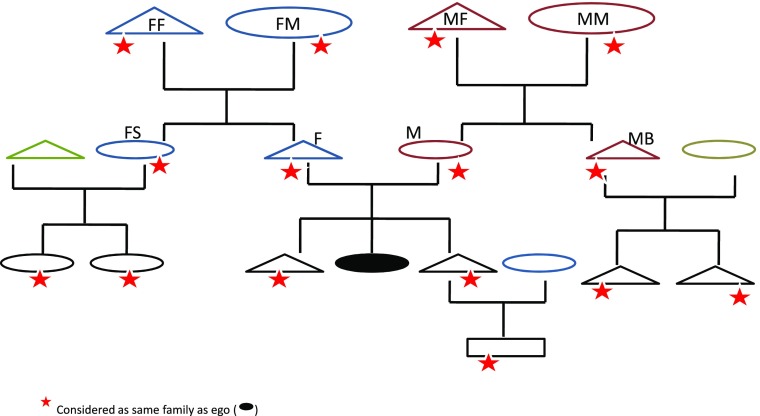
Hypothetical genealogy. Triangles represent males, and ovals, females; the element in black is the ego, and triangles and ovals with a star are considered members of the same family as ego

Prior to the analysis, we coded information regarding the interviewed child’s companions during the activities reported. We used census data to derive the sex, age, and kinship relation of every individual that accompanied respondents. To classify kinship relations, we differentiated between individuals from the same extended family as the respondent and those from others. The hypothetical extended family genealogy presented in Fig. 1 fits with the Baka cultural kinship system.

To evaluate the potential occurrence of vertical, oblique, and horizontal cultural transmission, we linked the age of the child to that of group members. Here we narrow the definition of transmission pathways as follows: vertical transmission refers to transmission from a parent or grandparent to a child, oblique transmission to transmission from adults other than (grand)parents to a child, and horizontal transmission refers to transmission among children (max. age 16 years). We coded each companion as (a) parent, (b) adult other than parent, (c) older child (by at least one year), (d) same-age child (within one year of age), and (e) younger child (by at least one year). All the children of the community have two parents.

We present descriptive statistics to show overall patterns of (co-)participation in activities and statistical random-effect models to test associations between individual attributes and types of events, on the one hand, and group size and composition, on the other. For descriptive purposes, we divided child respondents into three main age categories, drawing on previous work (Brisson 2010; Joiris 1998) and our own ethnographic data: (1) middle childhood, called *ngùmànàbo*; (2) preadolescence, called *lìngì* for boys and *sia* for girls; and (3) adolescence, called? *èwanjo* for boys and *sia* for girls. In the random-effect models, however, we used age as a continuous variable, taking age at baseline as the age of the respondents.

As part of our first goal, we also explored children’s group composition by projecting visualizations of children’s social networks during hunting, gathering, fishing, and agriculture. Although the descriptive and statistical analyses give detailed information about companions and group composition from the perspective of individuals, they lack a “bird’s-eye view” of the overall village social structure. To explore the cohesion of the network and the centrality of given individuals in the network, we also prepared visualizations of the social networks of co-presence during the four activities. To do this, we converted the data to a two-mode network in the form of a person-to-activity edge list, representing which individuals (respondents and nominees) were present during each unique activity. We then converted this edge list to a one-mode network in the form of a person-to-person edge list, representing the number of events in which pairs of individuals were co-present in the data set. We performed these calculations for each type of subsistence activity separately and then for the four activities combined. We show the networks based on all waves of interviews, where the edge width represents the number of unique events in which two people were present. In the visualizations of these weighted matrices, the edge width also depends on the number of interviews in which a child participated. Social network data were prepared and analyzed with IBM SPSS Statistics (24.0) and visualized with Visone (Brandes and Wagner 2004).

To understand how patterns were associated with attributes of events and individuals, we performed random-effects analysis—more precisely, generalized linear mixed model (GLMM) analyses—to control for the nested structure of the data since some individuals participated more often and reported more events than others. In these analyses, the data about events (*N* = 747) were hierarchically nested within the individuals who reported about the events (*N* = 58). Higher levels of nesting (e.g., based on clan) were discarded because the between-group variance at this level was negligible. For the 14 events that were reported by 2 or 3 informants, we used corresponding weights for the analysis (0.5 and 0.33, respectively). For categorical dependent variables, the linear model that was chosen was a multinomial logistic regression; for binary variables, we used binary logistic regressions, and for count data negative binomial regression with a log link function. For convergence, we excluded the five cases that reported only a single event from the GLMM analyses, so the analysis are based on *N* = 53 individuals. All continuous explanatory variables (age, number of siblings, and group size) were standardized. All models containing main effects improved in fit as evaluated from the Akaike Information Criterion when compared with empty models, but models containing interaction effects usually did not, and therefore interactions are presented in only one of the tables included below.

Our statistical analysis was structured around the three main goals of this work. Thus, we evaluated the number and characteristics of models available to children while performing subsistence activities as a way to evaluate whether knowledge flows differently across different activities. Specifically, we focused on (1) the frequency with which an activity was performed by a child alone; (2) the average group size when the activity was not performed alone; and (3) the group composition in terms of age, sex, and kinship. Explanatory variables were the type of event (objective 1) and the individual’s age (objective 2), and gender and the number of siblings (objective 3). Number of siblings was used to control for differences in household size, the type of activity, and, for some dependent variables, group size per event.

## Results

Of the 766 events related to subsistence activities that were reported during the 177 interviews, 747 were unique events (14 events were reported by two or three children): 488 hunting, 90 gathering, 32 fishing, and 137 agricultural events. Hunting events clearly stood out in number, but a large part of them were of short duration (e.g., visiting snares). 61.7% of all activities were performed alone, and 38.3% in dyads or groups (accompanied by parents or grandparents in 5.1%, by other adults in 7.2%, and only by other children in 26.0% of the cases).

### Variation in Group Composition among Subsistence Activities

Of the four activities considered, hunting occurred least often in a group, with 77.0% of the events done alone. In contrast, agriculture, gathering, and fishing were more often conducted in groups (only 52.4%, 12.8%, and 12.1%, respectively, were performed alone) (Table 1). The contrast between hunting and the other activities is confirmed in the statistical analysis, controlling for other individual-level variables (Table 2).

**Table 1:** Children’s group size

	Activities									
	Hunting		Gathering		Fishing		Agriculture		Overall	
	Girls	Boys	Girls	Boys	Girls	Boys	Girls	Boys	Girls	Boys
*N* reported events	112	384	48	46	20	13	60	83	240	526
*%* events when alone	77.0		12.8		12.1		52.4		61.7	
	83.9	75.0	10.4	15.2	5.0	23.1	55	50.6	55.4	64.6
Average number of people in group when not alone	3.2		3.6		3.8		4.5		3.7	
	3.7	3.1	4.0	3.3	3.9	3.6	5.6	3.8	4.3	3.3
(SD)	(1.6)	(1.2)	(1.4)	(1.2)	(1.7)	(1.3)	(2.0)	(1.8)	(1.8)	(1.4)

**Table 2:** Generalized linear mixed model predicting the type of company, using multinomial logistic regression (Reference category: Performing activity alone)

Parameter	With at least a parent or grandparent			With at least an adult other than parent or grandparent			With children only: older and same-age children			With children only: younger and/or same-age children		
	Coeff.	95% CI		Coeff.	95% CI		Coeff.	95% CI		Coeff.	95% CI	
		Lower	Upper		Lower	Upper		Lower	Upper		Lower	Upper
Fixed effects												
Intercept	−6.269^***^	−8.361	−4.176	−3.721^***^	−4.650	−2.793	−1.798^***^	−2.293	−1.303	−3.910^***^	−4.921	−2.899
*N* of siblings	−0.285	−0.741	0.170	−0.187	−0.545	0.170	−0.231	−0.484	0.023	−0.020	−0.403	0.362
Sex:^a^ Male	0.033	−0.841	0.907	−0.641	−1.343	0.061	−0.098	−0.646	0.449	1.653^**^	0.612	2.694
Age	−0.722^**^	−1.191	−0.252	0.155	−0.202	0.512	−0.408^**^	−0.673	−0.144	−0.044	−0.422	0.335
Activity^b^												
Gathering	6.061^***^	3.893	8.229	4.041^***^	2.895	5.186	2.984^***^	2.242	3.726	2.959^***^	2.038	3.880
Agriculture	5.025^***^	2.970	7.080	2.665^***^	1.696	3.633	0.239	−0.417	0.896	0.055	−0.889	0.999
Fishing	6.161^***^	3.711	8.611	4.807^***^	3.365	6.249	2.730^***^	1.520	3.940	2.291^*^	0.489	4.093
Random effects												
Level 2 var.	0.270	0.012	6.129	0.065	<.001	65.251	0.155	0.026	0.915	0.469	0.135	1.627
Level 1 var.	1 (fixed)											
Akaike IC	14,535.794											
% Correct	68.1%											

*N* = 761. ^*^p < .05; ^**^p < .01; ^***^p < .001

^a^(ref. = Female)

^b^(ref. = Hunting)

Excluding the events in which the informant reported being alone, the average number of people accompanying the respondent during subsistence activities was 2.7 (thus, groups had an average size of 3.7). Group size varied significantly depending on the activity. More specifically, larger groups were reported for agriculture (average 4.5 people) than for hunting (3.2). This difference is significant at *p* < .01, even after controlling for individual-level variables (see ESM Table S1). Gathering had an average group size of 3.6, and fishing, 3.8 members.

**Fig. 2:**
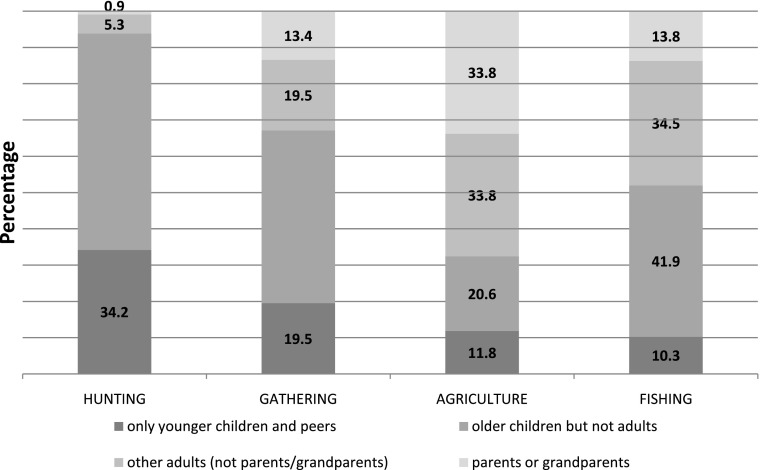
Children’s group composition, in percentages, calculated for the total number of reported events performed with others

Overall, the composition of the groups also varied according to the activities. Except for agriculture and girls’ fishing, the majority of events were performed in groups of children only (Fig. 2). For most activities, these groups involved children who were older than the interviewed children—as well as peers and younger children. In fewer cases, children were accompanied only by peers and younger children.

Figure S1 in the ESM shows that children’s social networks varied depending on the activity. For instance, in the case of fishing, subgroups were not connected one to another, implying that the total number of individuals with whom a child might interact was lower than for other activities in which all groups were connected. The analysis of group members in the cliques observed in the networks also suggested that children’s group composition varied from one activity to another. In other words, our results showed that children engaged with different individuals across different events, including individuals of different age categories. However, overall, the networks for all activities were mostly one large component, meaning that children varied their companions over time and activities. Thus, they were all directly or indirectly connected, which has implications for possible knowledge flows in the village.

We also found common patterns in all the activities: Fig. S1 shows that adults (orange tones) tended to appear at the periphery of children’s groups (green tones). This was the case for hunting, gathering, and fishing, for which, apparently, the same adults hardly ever interacted with more than one group of children. In all cases, adults had narrower edges with children than children had among themselves, implying that adults were mentioned less often than children. In contrast, while farming, adults accompanied children’s groups more often. Indeed, from our participant observation, we know that children often joined adults to farm, mostly groups of women. Another commonality across activities was that adults in children’s groups were indeed mostly women.

### Age Variations

Next, we investigated whether the networks described above show variation in terms of the age of the individuals forming the groups. Overall, children engaged with their parents or grandparents in 13.3% of the events. Indeed, Baka adults do not expect their children to accompany them during their productive activities, and children show a high degree of autonomy in choosing their daily activities. Parents and grandparents were almost absent from children’s hunting activities (0.3%), but their presence was more common for agriculture (33.8%). Controlling for the other variables and the nested structure of the data with GLMM, we found a significant difference in the group composition between hunting, on the one hand, and gathering, agriculture, and fishing, on the other (Table 2). Moreover, we found significant age-related differences regarding both adults’ presence and companion’s age (Table 2), with the likelihood of being accompanied by their parent(s) or grandparent(s) decreasing with children’s age (Table 2).During middle childhood and preadolescence, children were accompanied by their parents or grandparents in 38.5 and 43.6% of the events, respectively, whereas in adolescence, the percentage dropped to 17.9.

Second, overall, children engaged slightly more frequently with other adults than with their parents: in 18.8% of the events, they were accompanied by other adults *in the absence of* their own parents or grandparents. This was most often the case while farming (33.8%) and fishing (34.5%) (for the statistical associations see ESM Table S2), compared with gathering (19.5%) and hunting (5.3%). The presence of an adult other than a parent was rather stable across the three children’s age categories (Table 2).

Consequently, during most events performed in groups, children were only accompanied by other children (67.9%), particularly during hunting (93.2%). Indeed, according to our informants, adult hunters only invite boys to accompany them on hunting expeditions once they have the dexterity and discretion needed for successful hunting. In contrast, children were most often accompanied by adults for agricultural activities (32.4%) (see also Table S2).Overall, the frequency of children-only events tended to decrease with age, but this was mostly due to the significant decrease in the number of events with older children (Table 2), who were gradually replaced by adults (even though this coefficient was not significant). The latter category includes people aged 17 years and older, so this result can be attributed to the age categories employed rather than to a shift in possible pathways of knowledge transmission.

### Gender and Kinship Variations

Some of the patterns described thus far vary with regard to gender. In particular, girls reported significantly fewer hunting and more fishing and gathering events than boys (ESM Table S1). Girls also performed fewer subsistence activities alone (55.4%) than boys did (64.6% of the events; the difference is not directly visible in Table 2 because “boys” is the reference category). Furthermore, when they were accompanied, girls were more often with their parents, other adults, or same-age and older children (ESM Table S2), whereas boys were more often accompanied by same-age and younger children or only by same-age children. Also, girls were in slightly larger groups than boys (Table 3). Gender interactions with type of activity were mostly not significant (and therefore interactions were not included in the calculations).

**Table 3:** Generalized linear mixed model for the number of persons accompanying a child (for events in company), using negative binomial regression

Parameter.	Number of group members		
	Coeff.	95% CI	
		Lower	Upper
Fixed effects			
Intercept	1.366^***^	1.209	1.522
*N* of siblings	−0.030	−0.098	0.039
Sex:^a^ Male	−0.225^**^	−0.371	−0.080
Age	0.034	−0.036	0.105
Activity:^b^ Gathering	0.008	−0.163	0.180
Agriculture	0.232^**^	0.062	0.402
Fishing	0.075	−0.157	0.308
Random effects			
Level 2 variance	0.005	0.000	0.185
Level 1 variance	1(fixed)		
Akaike IC	360.709		

*N* = 288. ^*^p < .05; ^**^p < .01; ^***^p < .001

^a^(ref. = Female)

^b^(ref. = Hunting)

Overall, children were usually accompanied by same-sex individuals. 67.9% of all the events carried out in dyads or groups were exclusively composed of same-sex others, 22.5% in mixed-sex groups, and 9.6% in dyads or groups in which the interviewed child interacted only with individuals of the other sex. Larger groups were more likely to be mixed, and mixed-sex groups became less common with age, all else being equal (Table 4). The main effects of gender and type of activity and the interaction effects in Table 4, Model 2, suggest that girls were more likely to participate in mixed-sex groups during hunting and least likely during fishing, whereas the opposite was true for boys—in agreement with the predominant gender for the different activities. In this case, the Akaike Information Criterion improved with the inclusion of the interactions.

**Table 4:** Generalized linear mixed model for group composition with regard to gender (for events in company), using multinomial logistic regression (Reference category: Only same sex)

Parameter	Model 1 (main effects)						Model 2 (with interactions)					
	Mix of sexes			Only other sex			Mix of sexes			Only other sex		
	Coeff.	95% CI		Coeff.	95% CI		Coeff.	95% CI		Coeff.	95% CI	
		Lower	Upper		Lower	Upper		Lower	Upper		Lower	Upper
Fixed effects												
Intercept	−1.403^**^	−2.291	−0.514	−4.558^***^	−6.176	−2.939	−0.251	−1.459	0.957	−2.301^*^	−4.541	−0.062
*N* of siblings	−0.079	−0.509	0.351	−0.382	−0.995	0.231	−0.102	−0.565	0.361	−0.412	−1.065	0.241
Sex:^a^ Male	−0.517	−1.412	0.378	1.177	−0.280	2.635	−2.055^**^	−3.534	−0.576	−1.363	−3.881	1.156
Age	−0.636^**^	−1.097	−0.175	−0.163	0.624	−0.819	−0.655^**^	−1.146	−0.164	−0.151	−0.837	0.534
Activity^b^												
Gathering	0.722	−0.149	1.592	1.613^*^	0.310	2.916	−0.512	−1.855	0.830	−1.299	−4.324	1.726
Agriculture	0.711	−0.254	1.676	2.271^***^	0.966	3.577	−0.782	−2.331	0.766	−0.322	−3.531	2.887
Fishing	0.338	−0.850	1.526	2.062^*^	0.320	3.803	−1.244	−2.941	0.453	−0.364	−3.412	2.683
Group size	0.707^***^	0.336	1.078	−0.630	−1.388	0.078	0.745^***^	0.363	1.127	−0.575	−1.301	0.152
Interactions												
Gath. × Male							1.726	−0.073	3.525	3.366^*^	0.030	6.701
Agri. × Male							2.208^*^	0.276	4.139	2.965	−0.516	6.445
Fish. × Male							2.798^*^	0.307	5.288	2.968	−0.767	6.703
Random effects												
Level 2 var.	0.635	0.159	2.542	1.057	0.298	3.746	0.855	0.206	2.808	1.326	0.411	4.282
Level 1 var.	1(fixed)						1(fixed)					
Akaike IC	2505.618						2496.467					
% Correct	72.2%						75.7%					

*N* = 288. ^*^p < .05; ^**^p < .01; ^***^p < .001

^a^(ref. = Female)

^b^(ref. = Hunting)

With regard to family-related variations, we found that 20.1% of the reported events were performed with family members only, 28.3% of the activities were performed in groups that contained both family and non-family members, and 51.5% of the activities in dyads or groups were performed with only non-family members. Not surprisingly, larger groups were more likely to be mixed. The frequency of events formed with family members tended to be similar across the four activities (Table 5). Additional data (not shown) suggested that the most common family members were siblings and cousins. Age and gender were not significantly related to the presence of family when we controlled for the other variables.

**Table 5:** Generalized linear mixed model for group composition (for events in company), using multinomial logistic regression (Reference category: Only same family)

Parameter.	Family and non-family			Only non-family		
	Coeff.	95% CI		Coeff.	95% CI	
		Lower	Upper		Lower	Upper
Fixed effects						
Intercept	−1.523^*^	−0.299	2.747	1.885^**^	−0.619	3.152
N. siblings	0.030	−0.473	0.533	0.087	−0.523	0.696
Sex:^a^ Male	−0.663	−1.842	0.515	0.317	−1.001	1.635
Age	0.210	−0.397	0.817	0.600	−0.077	1.276
Activity^b^						
Gathering	−0.338	−1.544	0.868	−0.182	−1.218	0.855
Agriculture	−1.208	−2.529	0.113	−1.347^*^	−2.453	−0.241
Fishing	0.182	−1.598	1.962	0.106	−1.559	1.771
Group size	2.354^***^	1.590	3.118	1.204^***^	0.494	1.914
Random effects						
Level 2 var.	0.261	0.009	7.545	2.116	1.031	4.345
Level 1 var.	1(fixed)					
Akaike IC	2502.118					
% Correct	78.1%					

*N* = 288. ^*^p < .05; ^**^p < .01; ^***^p < .001

^a^(ref. = Female)

^b^(ref. = Hunting)

## Discussion

We organize the discussion around the three main goals of this work. But before beginning the discussion, we need to point out some limitations inherent to the nature of the data. First, since our data come from only one village, which, in addition, is larger than many settlements found in other forager societies in the Congo Basin, we are not sure whether the observed patterns can be generalized. Second, we could only interview 61% of the children between 5 and 16 years of age, who were not randomly chosen; therefore, our sample might suffer from self-selection bias. Indeed, the youngest children are under-represented: of the 28 children who did not take part, 23 were between 5 and 6 years old. We do not know if children who participated varied in other, more nuanced ways from those who did not. Moreover, younger children might have been shier than older children, thus underreporting the people they were with, although we did test the reliability of the data children reported to us, even for the youngest ones. A participation rate of 61% of the total population is large enough for drawing conclusions on the individual level, and the number of groups reported by these individuals is a rich resource for understanding group composition. Nevertheless, we need to interpret our results with some caution because we cannot discard the possibility that self-selection biases affect the structure of the social networks produced here, particularly since some children were interviewed more often than others. We tried to assess the magnitude of this bias by running an analysis (not shown) in which we used only one observation per interviewed child and compared the results with the ones obtained when information from multiple interviews per child were used. Since the results were very similar, we chose to keep all the interviews in the reported analyses to avoid loss of information. Finally, this study was only aimed at assessing potential cultural transmission pathways, and thus it does not cover all the factors that might affect the dynamics of cultural transmission.

### Children’s Group Composition and Knowledge Flow

Children’s group composition varied depending on the activity performed, suggesting that knowledge might flow differently across activities. For example, boys engage in hunting activities frequently, but mostly alone. Moreover, when Baka boys hunt in groups, these groups are small and composed of children. Because the acquisition of hunting skills is a complex process that is not completed until adulthood (Duda et al. 2017; Gurven et al. 2006), the involvement of boys in hunting alone and in small groups can be seen as the ideal environment that allows children to practice the complex set of skills needed for adult hunting (MacDonald 2007; Ingold 2010; Dira and Hewlett 2016). Moreover, although children’s involvement in subsistence activities mostly depends on children’s choice, children are not allowed to join adults’ hunting trips until they have sufficient skills, an exclusion that does not obtain in any other subsistence activity.

The three other subsistence activities seem to favor social learning in different ways. Gathering is largely performed in children-only groups, with a different group composition from one event to another. Such group dynamism results in a large and dense network of interactions, which potentially allows the free flow of knowledge among all the children in a community. This might result in considerable homogeneity of gathering knowledge and practices, a hypothesis at least partly confirmed by empirical data from the BaYaka (Salali et al. 2016).

In contrast to gathering, agriculture shows a network formed by larger subgroups and characterized by more adult involvement. The presence of mixed-age groups might lead to the ample diffusion of agricultural knowledge, including adults’ knowledge.

Finally, fishing shows a smaller and more fragmented network, with subgroups being more isolated one from another, which potentially reduces the pool of role models from which to learn. During fishing, children often interacted with adults. From an early age, children participate in collective fishing expeditions in which they can learn simultaneously from their peers, older children, and adults (Gallois and Duda 2016).

In sum, the presence of mixed-age groups during children’s subsistence activities, notably visible here for agriculture and fishing, might allow children to acquire new knowledge from several sources simultaneously (Gray 2011; Garfield et al. 2016; Hewlett and Roulette 2016). This strategy might, indeed, be much more efficient than performing activities only with same-age children or even adults (cf. Hewlett 2016). Moreover, this pattern parallels the concerted mode of knowledge transmission, i.e., the transmission of knowledge from many individuals to one (who can be from the same generation or not), which has been identified as a factor fostering the conservation of cultural traits (Hewlett and Cavalli-Sforza 1986). Furthermore, the variation in group composition from one activity to another underlines the presence of multiple sets of resources for subsistence-related knowledge acquisition, which vary according to sex, age, kinship relation, and activity performed (i.e., domain of knowledge). Most importantly, we see that children are involved with different individuals according to the domain of activities, which may suggest that children might be learning different subsistence-related knowledge from different individuals. This result is in line with the hypothesis that the way information flows varies according to the activity. For some activities (e.g., hunting and gathering) preferred groups are small (involving few people) but fluid, with many connections between each subgroups that facilitate the homogeneous diffusion of knowledge within the community. For other activities (e.g., fishing), groups seem to more stable but less interconnected, potentially resulting in particular subgroups. And finally, for others (e.g., agriculture), groups seem to be larger and more fluid, which might allow the quick diffusion of knowledge among the whole community. Although these patterns are coherent with the observations collected during adults’ and children’s daily activities, further behavioral observation can better assess the actual transmission of knowledge during such activities.

### Pathways of Cultural Transmission from Middle Childhood to Adolescence

Two main findings of our work are that parents and grandparents engage only in 5% of their children’s subsistence activities, and that parent’s and grandparents’ presence decreases with children’s age. This might reflect both adult time allocation (as many subsistence activities occur outside the village) and parental encouragement of children’s autonomy (Lew-Levy et al. 2017; Whiting 1980; Whiting and Whiting 1975). Indeed, during fieldwork we often observed an adult around children’s groups; that is, children’s activities occurred within the visual or auditory range of adults, a situation that encourages children’s independent learning, while providing some level of parental supervision (Hewlett 1991a; Boyette 2013; Lew-Levy et al. 2017). Moreover, in accordance with what has been reported in other settings (Aunger 2000; Henrich and Broesch 2011; Migliano et al. 2017), children’s engagement with parents and grandparents tends to decrease with age, potentially allowing Baka adolescents to expand their set of models for knowledge acquisition to a broader network settled outside the close family.

Our findings lead to an important consideration. If subsistence-related knowledge is largely acquired while performing subsistence activities in which parents are only loosely involved, then children might need to rely on other role models for knowledge acquisition. We should clarify that our argument does not imply that parents do not have a role in knowledge transmission; parents are present in other settings also essential for knowledge transmission, as when performing songs and tales or telling stories about their daily experiences at night, as shown among the Baka (Fitzgerald 2011 and also in our own fieldwork) and other societies (e.g., Fernández-Llamazares and Cabeza 2018; Sugiyama 2017). However, our data suggest that when children engage in subsistence-related activities, the potential for vertical transmission, as measured by time spent with parents and grandparents, is lower than for oblique and horizontal transmission, a finding that may be consistent with recent results among other hunter-gatherer groups (Bird and Bliege Bird 2005; Boyette 2013; Garfield et al. 2016; Hewlett et al. 2011). Therefore, further studies are needed to explore how the transmission of knowledge occurs during such activities.

We also found that other adults were only present in 7% of the cases, even though they formed 45% of the population of the village, with some remarkable exceptions during agricultural activities and girls’ fishing. Furthermore, although previous research in hunter-gatherer societies suggests that oblique transmission increases with children’s age (Garfield et al. 2016), we found that it is not the case for all the subsistence activities explored in our study. Therefore, our findings also indicate that oblique transmission might not be predominant while children perform subsistence activities. However, because the distinction between oblique and horizontal pathways was only drawn on the basis of age (adults and children are differentiated at between 16 and 17 years of age), it would be worth exploring whether the patterns would change if the definition of age categories was changed. In the same vein, exploring gaps in age between individuals might yield insight into the definition of oblique transmission pathways.

An important implication of the rare presence of adults in children’s groups during subsistence activities is that knowledge may largely circulate among children. This is an important finding because it points out the potential contribution of children to social learning (Gallois et al. 2017; Harris 1995; Lancy 1996). In discussing this finding, we should go back to our definition of horizontal transmission—knowledge transmission between children. For the empirical work presented here, we considered the category “children” as static, but we acknowledge that this and the other age-based categories are fluid; two persons 16 and 20 years of age would be assigned to two different categories but are closer in age (and likely in maturity) than two individuals aged 5 and 16, who would be placed in the same category. Consequently, while acknowledging the importance of children in knowledge transmission, we also want to highlight that the analytical category “horizontal transmission” used here might mask important differences associated with the transmission of knowledge between children of different ages. Indeed, as our results show, children’s interactions with same-age or with older individuals would change according to the activity but also with the child’s age. In this sense, we need a better understanding of horizontal transmission, and expecially when, why, and how it occurs during childhood and adolescence.

In the same vein, in this study we considered horizontal transmission as the transmission of knowledge between individuals of the same generation, focusing only on the transmission occurring when children are sharing knowledge among themselves. However, since generations are not clear units and are “more of an intuitive notion than a well-defined quantity” (Bienvenu and Legendre 2015:834), it is difficult to determine who would be part of the same generation and who would not.

### Model-Based Biases

Despite the diversity of learning strategies reported in this work, one commonality stands out: irrespective of the children’s age, we found a strong gender homophily and a notable engagement with family members. Homophily might shed light on a sex-biased model of knowledge acquisition, as also reported in other settings (Henrich and Broesch 2011; Hewlett and Cavalli-Sforza 1986; Reyes-García et al. 2009), which might be accentuated in larger settings in which same-sex and same-age partners are easier to find (Hewlett 1991b).

We have shown elsewhere (Gallois et al. 2015) that Baka children present a gender division of subsistence activities similar to Baka adults, with girls being more involved in domestic tasks, agriculture, and fishing and boys more involved in hunting. Although children mostly perform activities without adults, we found that the social composition of children’s groups mirrors the sex differentiation of adults’ subsistence activities. Therefore from middle childhood onwards, children not only acquire subsistence-related knowledge, they also seem to acquire norms of social interactions and organization for the performance of subsistence activities, as has also been highlighted in other settings (Harris 1995; Lew-Levy et al. 2017; Wood et al. 2013).

Another important tendency revealed by our data is that children from the same family interact with each other frequently, a finding that has also been shown among the Agta and the BaYaka (Migliano et al. 2017). Although previous research shows that the transmission of ethnobotanical knowledge is largely shaped by kinship relations (Salpeteur et al. 2015), further studies are needed to understand to what extent the transmission of subsistence-related knowledge is shaped by kinship relations, and how it might vary depending on the knowledge domain. Furthermore, considering that a large amount of cultural knowledge is acquired before adolescence, it would be worth exploring who are the main family members that intervene in cultural transmission during childhood and how the interaction of kinship during knowledge acquisition might affect the intracultural distribution of subsistence-related knowledge among the Baka specifically and small-scale societies in general.

## Conclusion

Results from this research show that the structure of children’s social networks largely varies from one activity to another, resulting in variation in the potential role models from which children can learn by observation according to their age, sex, and the activity performed. This finding provides support for the idea that cultural knowledge is acquired through “multiple learning strategies” (Kline et al. 2013; Reyes-García et al. 2016; Schniter et al. 2015), thus helping move forward standard studies on the acquisition of cultural knowledge, which have heretofore been based on studying the importance of one or another type of model. Results of this research also suggest that knowledge acquisition during Baka children’s subsistence activities might mainly occur between children and between same-sex individuals. This finding highlights the importance of children as actively engaged social actors, not only in the acquisition, but also in the transmission of cultural knowledge.

## Electronic supplementary material

ESM 1 (PDF 751 KB)
