# Supplementary material for: Social Networks and Knowledge Transmission Strategies among Baka Children, Southeastern Cameroon
Source: Hum Nat. 2018 Oct 24;29(4):442–63. doi: 10.1007/s12110-018-9328-0 (PMC6208833; doi:10.1007/s12110-018-9328-0)
Supplement: Supplementary file 1 — (PDF 751 KB) [file 12110_2018_9328_MOESM1_ESM.pdf]

Electronic Supplementary Material
for

Social Networks and Knowledge Transmission Strategies among Baka Children, Southeastern Cameroon

Sandrine Gallois, Miranda J. Lubbers, Barry Hewlett, and Victoria Reyes-García

Human Nature 29(4), 2018. DOI: 10.1007/s12110-018-9328-0

Table S1. Generalized linear mixed model for the number of persons accompanying a child (for events in company), using negative binomial regression. $N = 288$. * $p < .05$; ** $p < .01$; *** $p < .001$.

**Parameter**	**Number of group members**		
	**Coeff.**	**95% CI**	
		**Lower**	**Upper**
Fixed effects			
Intercept	1.366***	1.209	1.522
Number of siblings	−0.030	−0.098	0.039
Sex			
Male	−0.225**	−0.371	−0.080
Age	0.034	−0.036	0.105
Activity			
Gathering	0.008	−0.163	0.180
Agriculture	0.232**	0.062	0.402
Fishing	0.075	−0.157	0.308
Random effects			
Level 2 variance	0.005	0.000	0.185
Level 1 variance	1 (fixed)		
Akaike IC	360.709		

Figure S1 (see following pages). Children’s social network during subsistence activities. Nodes represent individuals, and edges between two nodes indicate that activities were performed together. Nodes with black borders represent respondents; the other nodes indicate individuals who were mentioned by the respondents but were not interviewed. Isolated nodes have been excluded from the network. Node color and size represent individuals’ age categories, and shape indicates sex (see legend). Labels in the nodes refer to the household code. Varying edge width indicates the numbers of events in which participants were both present.

A: HUNTING

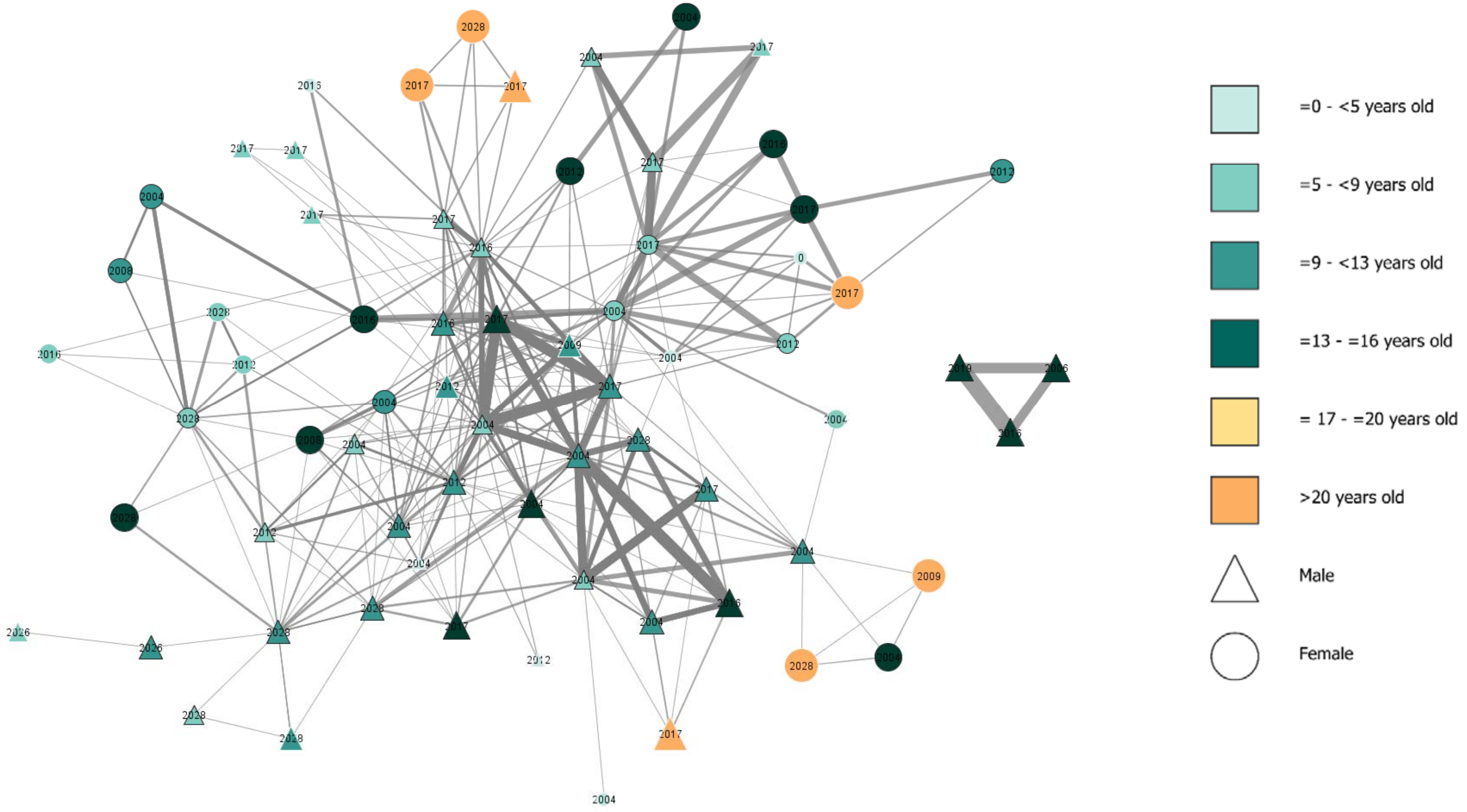

=0 - <5 years old
=5 - <9 years old
=9 - <13 years old
=13 - =16 years old
= 17 - =20 years old
>20 years old
Male
Female

B: GATHERING

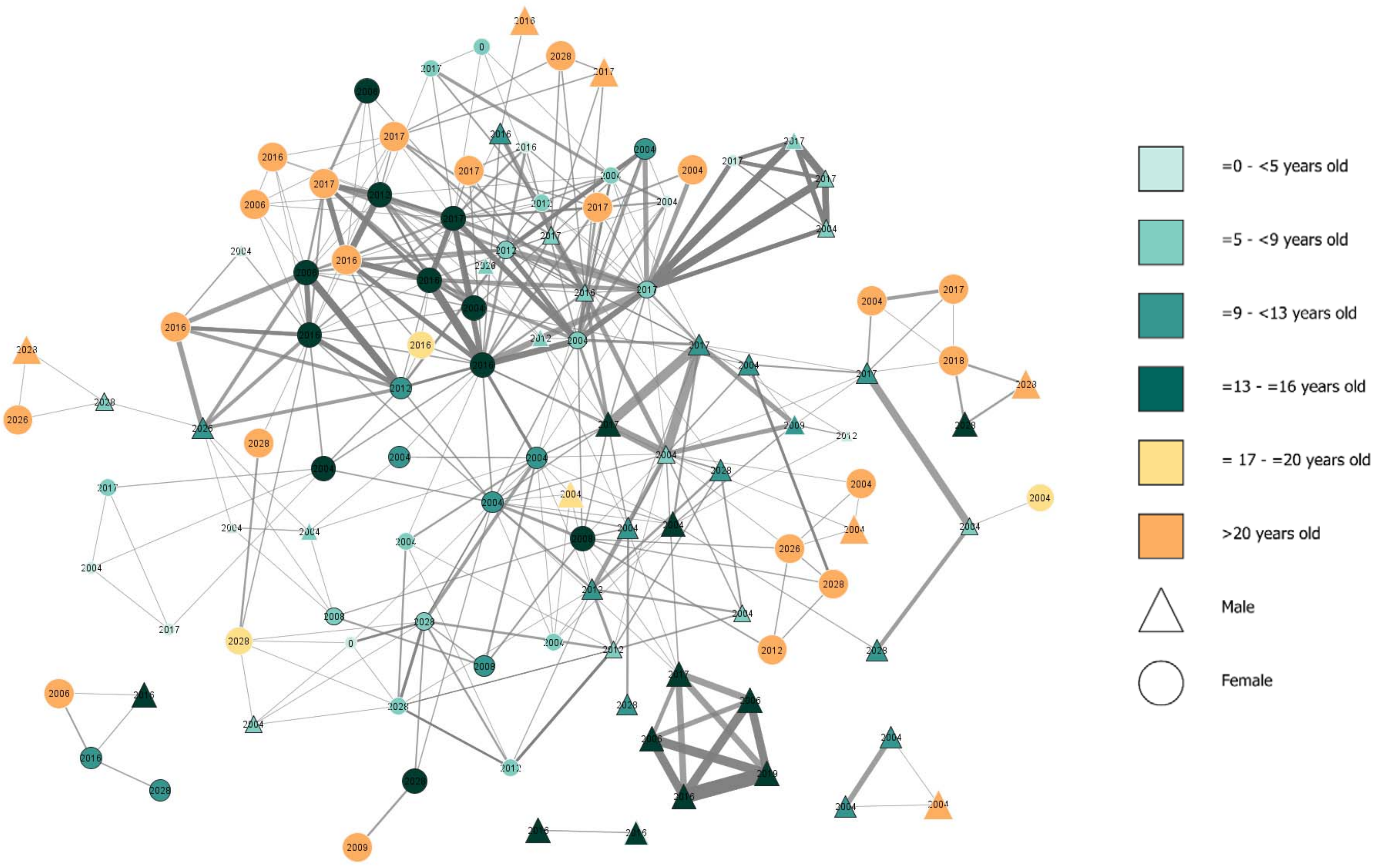

=0 - <5 years old
=5 - <9 years old
=9 - <13 years old
=13 - =16 years old
= 17 - =20 years old
>20 years old
Male
Female

C: AGRICULTURE

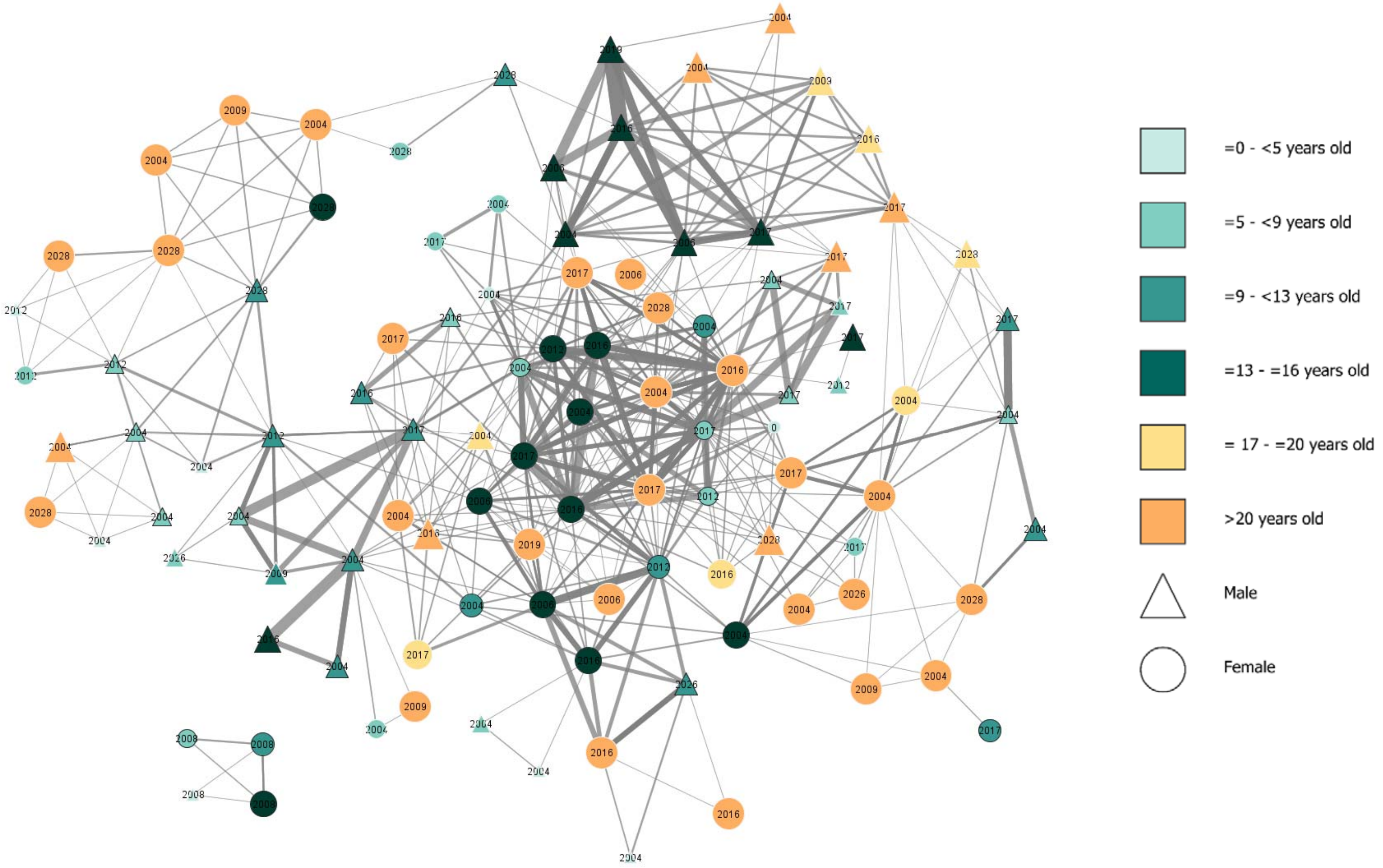

D: FISHING

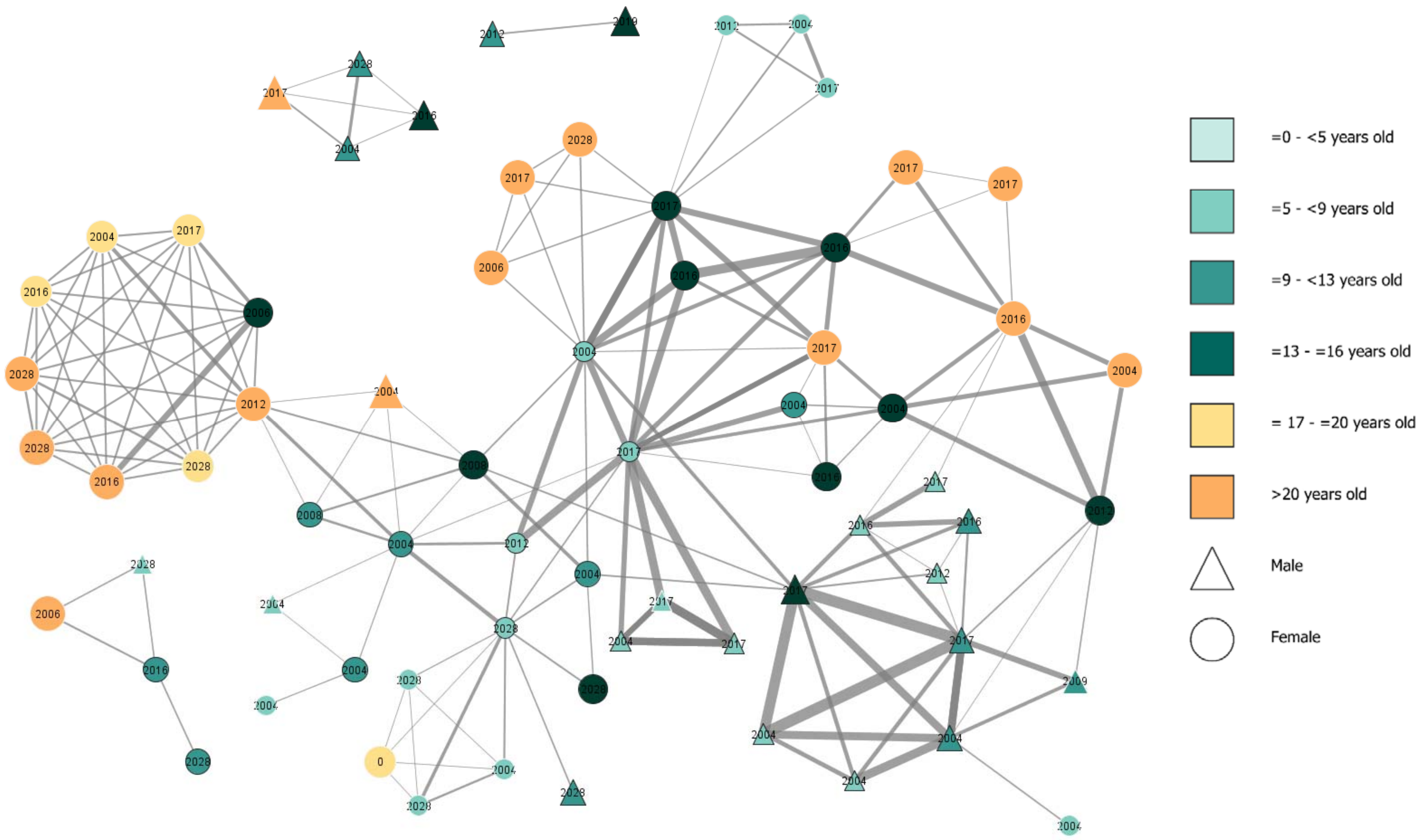
=0 - <5 years old
=5 - <9 years old
=9 - <13 years old
=13 - =16 years old
= 17 - =20 years old
>20 years old
Male
Female

Table S2. Generalized linear mixed model predicting the type of company, using multinomial logistic regression, only for events performed in company (Reference category: With peers or younger children, or only peers). $N = 288$. $^{*}\ p < .05$; $^{**}\ p < .01$; $^{***}\ p < .001$

**Parameter**	**With parents or grandparents (and possibly others)**			**With other adults, but not parents or grandparents (but possibly with others)**			**With peers and older children only**		
	**Coeff.**	**95% CI**		**Coeff.**	**95% CI**		**Coeff.**	**95% CI**	
		**Lower**	**Upper**		**Lower**	**Upper**		**Lower**	**Upper**
Fixed effects									
Intercept	−2.441*	−4.791	−0.091	0.189	−1.182	1.560	2. 249***	1.105	3.393
N. siblings	−0.352	−0.953	0.249	−0.252	−0.775	0.270	−0.263	−0.726	0.200
Sex									
Male	−1.876**	−3.279	−0.473	−2.342***	−3.574	−1.109	−1.918**	−3.106	−0.730
Age	−1.019**	−1.684	−0.353	−0.030	−0.587	0.526	−0.514*	−1.020	−0.009
Activity									
Gathering	3.227**	0.998	5.457	1.073	−0.207	2.353	−0.041	−0.931	0.849
Agriculture	5.480***	3.162	7.797	2.754***	1.346	4.163	0.166	−1.023	1.355
Fishing	3.858**	1.119	6.596	2.388*	0.515	4.260	0.144	−1.537	1.826
Random effects									
Level 2 var.	0.708	0.108	4.620	0.415	0.058	2.999	0.864	0.340	2.198
Level 1 var.	1 (fixed)								
Akaike IC	3572.457								
% Correct	64.2%								
